# Gut microbiota was highly related to the immune status in chronic obstructive pulmonary disease patients

**DOI:** 10.18632/aging.205532

**Published:** 2024-02-12

**Authors:** Mei Wang, Jun Song, Huizhen Yang, Xiaoyu Wu, Jin Zhang, Sheng Wang

**Affiliations:** 1 Department of Respiratory, Zhejiang Jinhua Guangfu Cancer Hospital, Jinhua 321000, Zhejiang, China; 2 Department of Psychiatry, Jinhua Second People’s Hospital, Jinhua 321000, Zhejiang, China

**Keywords:** chronic obstructive pulmonary disease, gut microbiota, immunologic factors, immune status, IFscore

## Abstract

This study aimed to explore the profile of gut microbiota and immunological state in COPD patients. 80 fecal and blood samples were collected from 40 COPD patients and 40 healthy controls (HC) and analyzed with 16s-rRNA gene sequencing and immunofactor omics analysis to investigate the profile of gut microbiota and immunologic factors (IFs). The linear discriminant analysis (LDA) effect size (LefSe) was used to determine the biomarker’s taxa. The random forest and LASSO regression analysis were executed to screen IFs and develop an IFscore model. The correlation between gut microbiota and IFs, along with the IFscore and the diversity of gut microbiota, was evaluated with the Spearman analysis. The α and β diversity showed that the composition and distribution of gut microbiota in the COPD group differed from that of the HC group. 7 differential taxa at the phylum level and 17 differential taxa at the genus level were found. LefSe analysis screened out 5 biomarker’s taxa. 32 differential IFs (up-regulated 27 IFs and down-regulated 5 IFs) were identified between two groups, and 5 IFs (CCL3, CXCL9, CCL7, IL2, IL4) were used to construct an IFscore model. The Spearman analysis revealed that 29 IFs were highly related to 5 biomarker’s taxa and enriched in 16 pathways. Furthermore, the relationship between the IFscore and gut microbiota diversity was very close. The gut microbiota and IFs profile in COPD patients differed from that in healthy individuals. Gut microbiota was highly related to the immune status in COPD patients.

## INTRODUCTION

Chronic obstructive pulmonary disease (COPD) is one of the most common lung diseases, characterized by chronic airway obstruction, chronic inflammation, and a progressive and irreversible decline in lung function [1, 2]. The World Health Organization reports that COPD is the third leading cause of death and causes over 3.3 million deaths globally [2–4]. Tobacco use, genetic factors, environmental pollution, and infections are the major risk factors for COPD [5]. Due to those risk factors, the total mortality rate of COPD increased by 14.1% between 2009 and 2019 [2]. The high morbidity and mortality and low quality of life led by COPD have resulted in severe economic and social burdens [6]. Therefore, it is urgent to identify more underlying pathophysiological mechanisms of COPD development that can be effectively influenced for therapeutic purposes.

The gut microbiota refers to the microbial community living in the gut, including bacteria, fungi, protozoa, and viruses. In addition to affecting metabolic, immune, and endocrine systems, gut microbiota influences the functions of extra-gut organs and disease development [7–9]. For this reason, researchers have proposed the theory of the “Gut-lung axis,” meaning the long-distance cross-talk between lung and gut. Notably, increasing studies have linked the alterations in the gut microbiota and the pathogenesis and development of COPD [10]. Research has proved that the composition and distribution of gut microbiota in COPD differs from those of healthy individuals [10–13]. Additionally, the abnormal gut microbiota in COPD is correlated to airway inflammation level, and the progression of COPD in mice is accelerated by fecal transplantation from COPD to mice [14], hinting at a direct influence of the gut microbiota on COPD.

Chronic exposure to risk factors triggers and exacerbates the development of COPD by inducing airway inflammation and immune cell infiltration into both the central airways, distal airways, and lung parenchyma [15]. Inflammation and immune cell infiltration in the bronchial tree are considered the essential pathogenesis of COPD [16]. Inflammatory and immune cells release various immunologic factors (cytokines, chemokines, and mediators). These immunologic factors (IFs) can be divided into two types: 1) pro-inflammatory: TNF-α, IL-1, IL-13, IL-6, and so on; 2) anti-inflammatory: IL-10, TGF-β, and so on [17, 18]. An imbalance of IFs would cause persistent immune cells to infiltrate and aggravate the progressive destruction of the lung in COPD by releasing destructive enzymes [19].

In the study, we evaluated the relationship between gut microbiota and systemic immune factors for the first time. Firstly, we applied 16s-rRNA gene sequencing to analyze the composition and distribution of gut microbiota in COPD. Then, we take advantage of immunofactor omics analysis to investigate the profile of 48 IFs. Finally, we explored the relationship of gut microbiota to IFs and developed an IFscore with 5 IFs to evaluate the immune levels and their correlation with gut microbiota.

## RESULTS

### Alterations in α and β diversity between healthy controls (HC) and COPD

**Figure 1:**
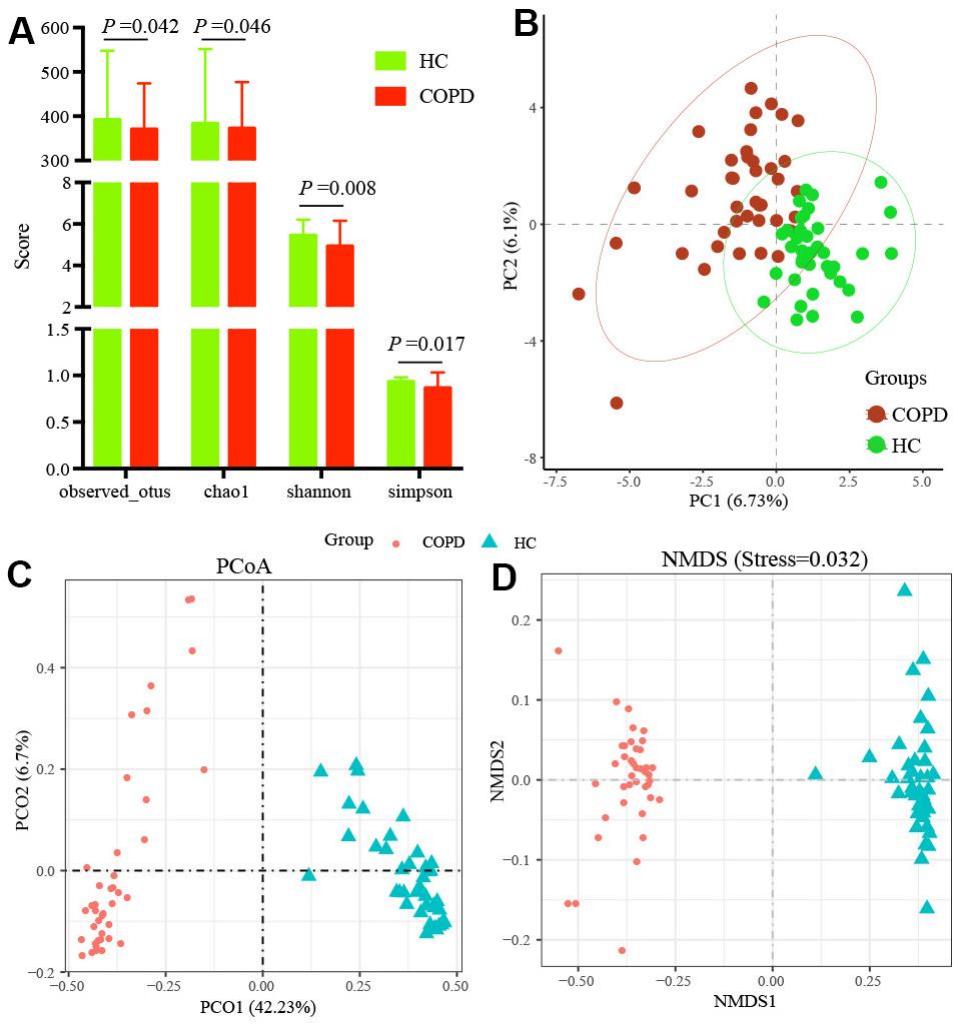
**The α and β diversity assessment of gut microbiota.** (**A**) The α diversity was evaluated by observed OTUs, Chao1, Shannon and Simpson indexes. The β diversity was evaluated by (**B**) PCA, (**C**) PCoA and (**D**) NMDS. H: healthy controls; COPD: Chronic obstructive pulmonary disease; PCA: Principal Component Analysis; PCoA; Principal coordinate analysis; NMDS: Non-metric multidimensional scaling.

**Figure 2:**
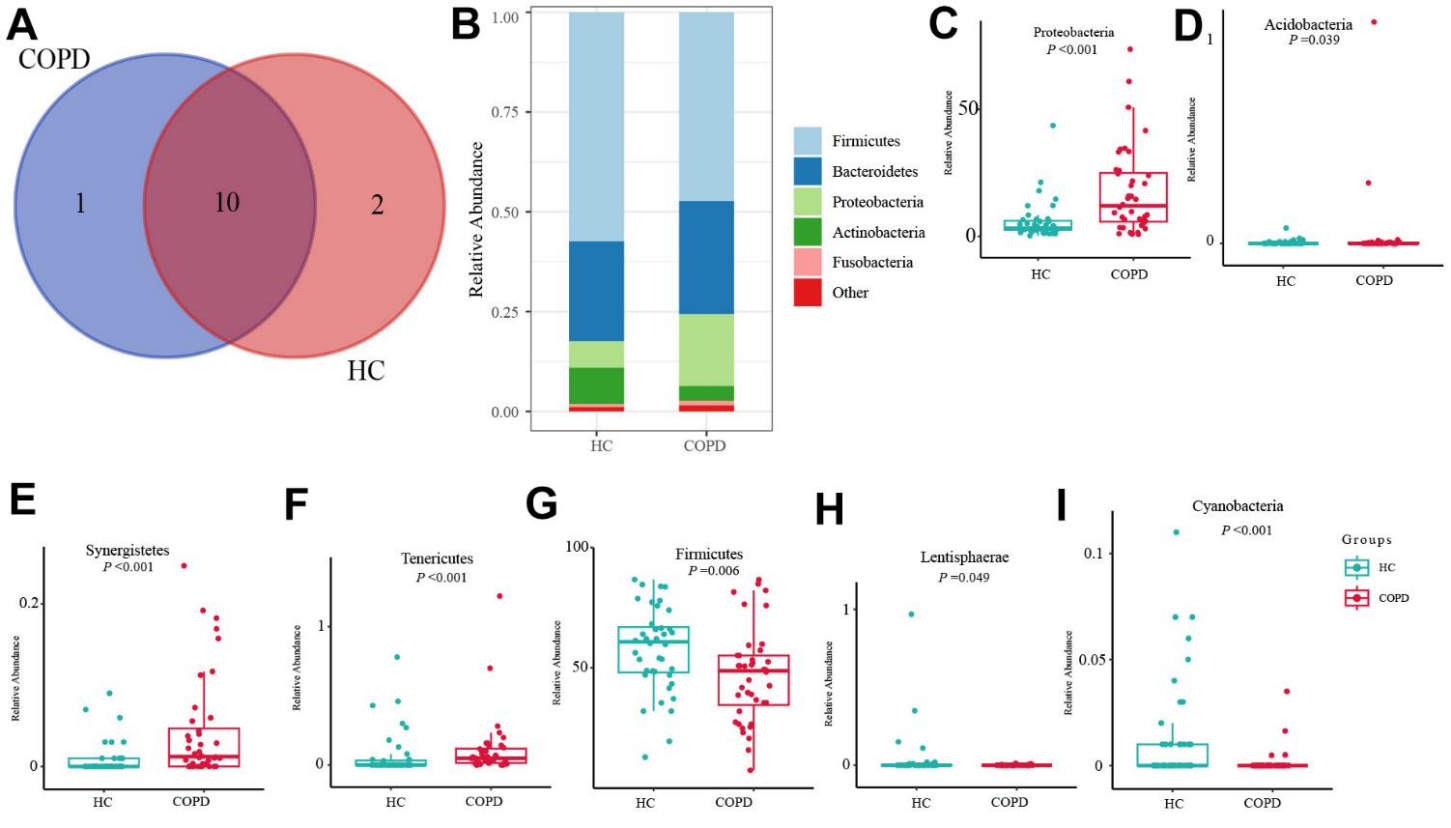
**Taxonomy comparison of gut microbiome at phylum level.** (**A**) Phyla identified in HC and COPD group. (**B**) Top 5 phyla in two groups. (**C**–**I**) Differential phyla between two groups.

We analyzed 80 stool samples (HC: 40; COPD: 40) and obtained 7 752 964 raw reads mapped to 7364 OTUs. The observed OTUs, Chao1, Shannon, and Simpson index were calculated to evaluate the α diversity. All indexes in the COPD group were significantly decreased (observed OTUs P =0.042, Chao1 P= 0.046, Shannon P =0.008, Simpson P =0.017, Figure 1A). The PCA presented two groups clustered in independent regions (Figure 2B). The PCoA and NMDS showed samples in two groups displayed tighter clustering (Figure 1C, 1D).

### Taxonomic distributions

The taxonomic composition of gut microbiota at phylum, class, order, family, and genus levels was investigated. At the phylum level, 12 and 11 phyla were identified in the HC and COPD group, respectively (Figure 2A). The top three abundant phyla in the COPD group were Firmicutes, followed by Bacteroidetes and Proteobacteria (Figure 2B). In the COPD group, the top three abundant phyla were Firmicutes, followed by Bacteroidetes and Proteobacteria (Figure 2B). Compared with the HC group, the abundance of phylum-*Proteobacteris, Acidobacteria, Synergistetes Acidobacteria*, and *Tenericutes* increased in the COPD group. In contrast, the proportion of Firmicutes, *Lentisphaerae*, and *Cyanobacteria* was decreased (Figure 2C–2I).

**Figure 3:**
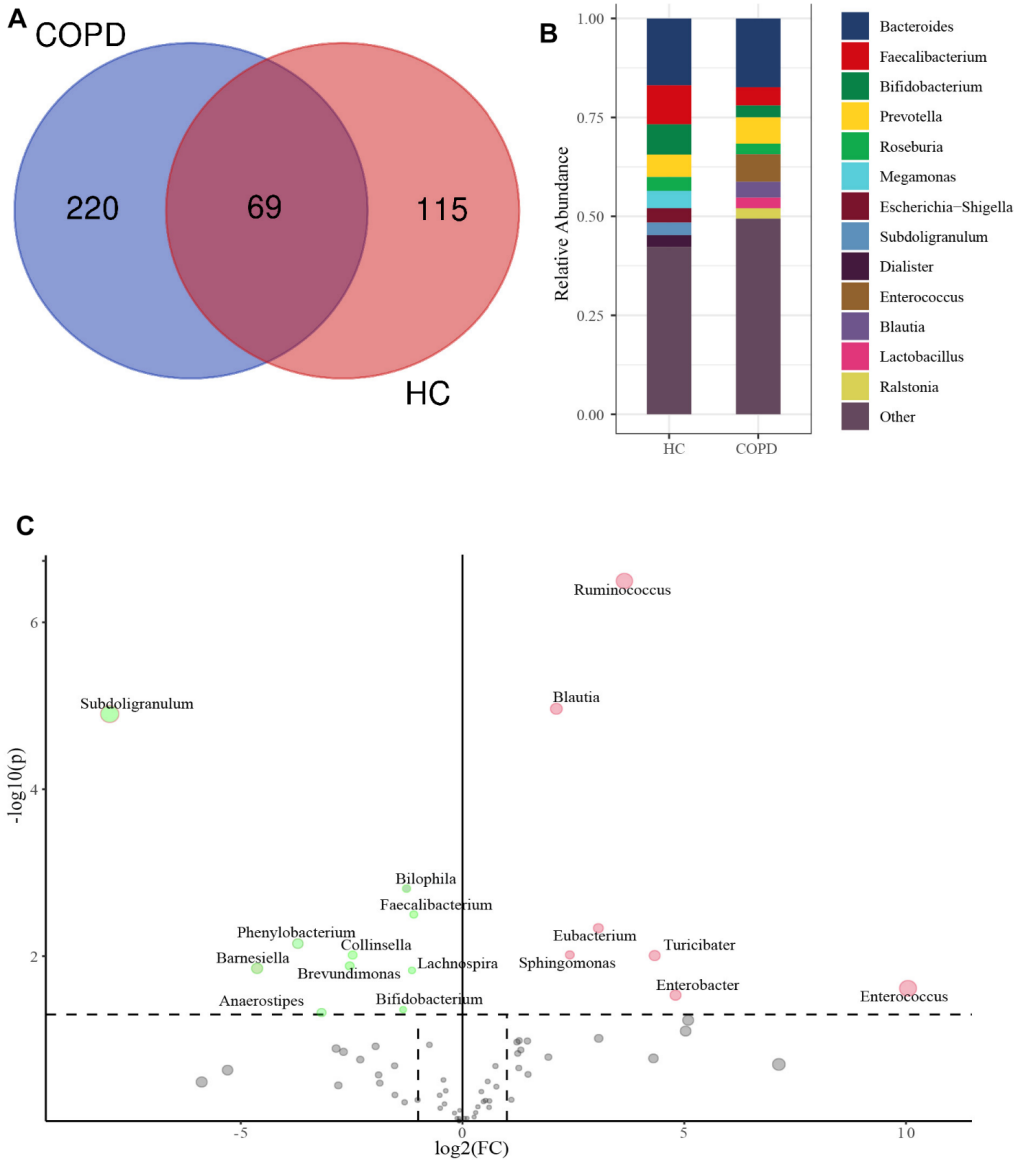
**Taxonomy comparison of gut microbiome at genus level.** (**A**) Genera identified in HC and COPD group. (**B**) Top 10 genera in two groups. (**C**) Differential genera between two groups. FC: Fold change.

At genus levels, 289 genera were identified in the COPD group and 184 in the HC group (Figure 3A). The top 10 genera in both two groups were shown in Figure 3B. The abundance of 7 genera in the COPD group was significantly higher than that in the HC group (Figure 3C). In addition, the proportion of 10 genera was substantially lower than that in the HC group.

### Identification of biomarker’s taxa in COPD group

**Figure 4:**
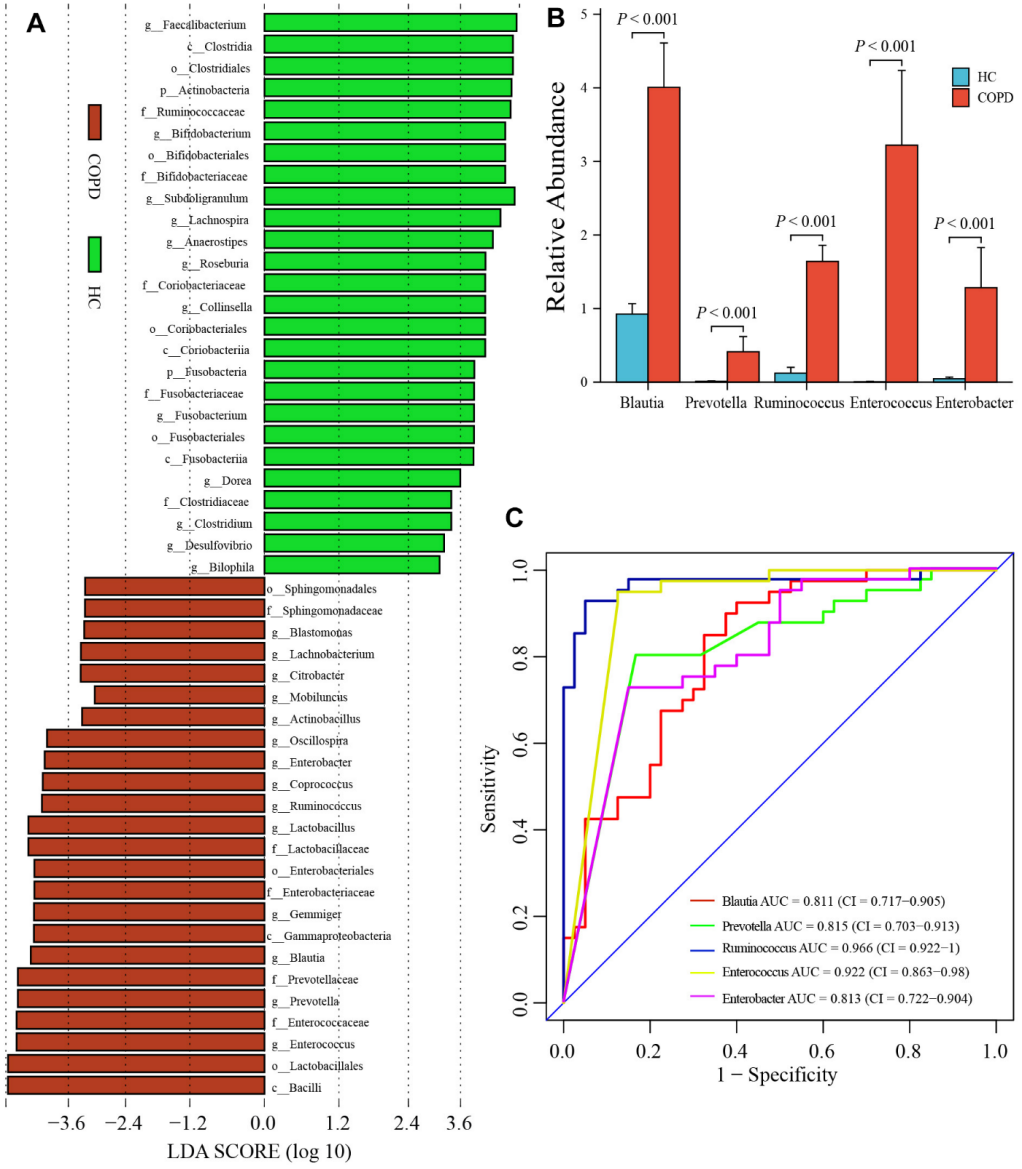
**Identification of biomarker’s taxa.** (**A**) LefSe determined 14 floras at genus levels were markedly enriched in the COPD group. (**B**) Wilcoxon rank-sum test showed the abundance of 5 floras was significantly increased in COPD group among 14 floras. (**C**) The ROC of 5 biomarker’s taxa. LefSe: The Linear discriminant analysis (LDA) effect size; ROC: The receiver operating characteristic curve; AUC, the area under the ROC.

The LefSe analysis revealed that 14 floras at genus levels were markedly enriched in the COPD group (LDA value> 3.0 and P< 0.01, Figure 4A). Then, the difference analysis between the two groups found that among 14 floras, the abundance of 5 floras in the COPD group was significantly increased (*Blautia*: P <0.001, *Prevotella*: P <0.001; *Ruminococcus*: P <0.001, *Enterococcus*: P <0.001, *Enterobacter*: P <0.001; Figure 4B). Hence, we selected those 5 genera as the biomarker’s taxa in COPD. The receiver operating characteristic curves (ROC) of 5 genera were presented in Figure 4C, and the area under the ROC (AUC) was 0.811, 0.815, 0.966, 0.922, and 0.813 for *Blautia, Prevotella, Ruminococcus, Enterococcus*, and *Enterobacter*, respectively.

### The influence of clinical indexes on the distribution of gut microbiota

**Figure 5:**
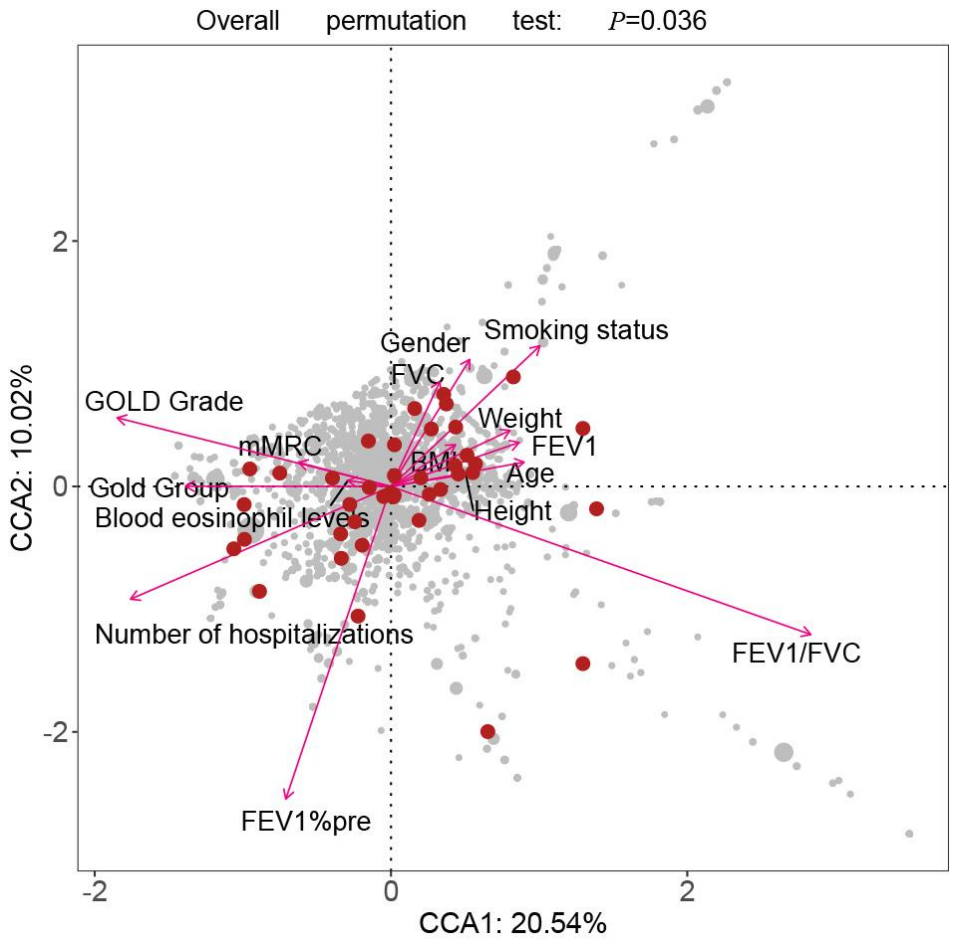
**The CCA analysis revealed the influence of clinical parameters on the distribution and structure of the gut microbiota.** CCA: The canonical correspondence analysis; GOLD: Global Initiative for Chronic Obstructive Lung Disease.

The CCA analysis showed that 6 factors had a vital impact on the distribution of the gut microbiota, including FEV1/FVC (P <0.001), FEV1%pre (P <0.001), GOLD Grade (P =0.008), COPD Group (P =0.024), smoking history (P =0.013), and frequency of hospitalization due to COPD at last year (P =0.006) (Figure 5).

### Functional annotation analysis

**Figure 6:**
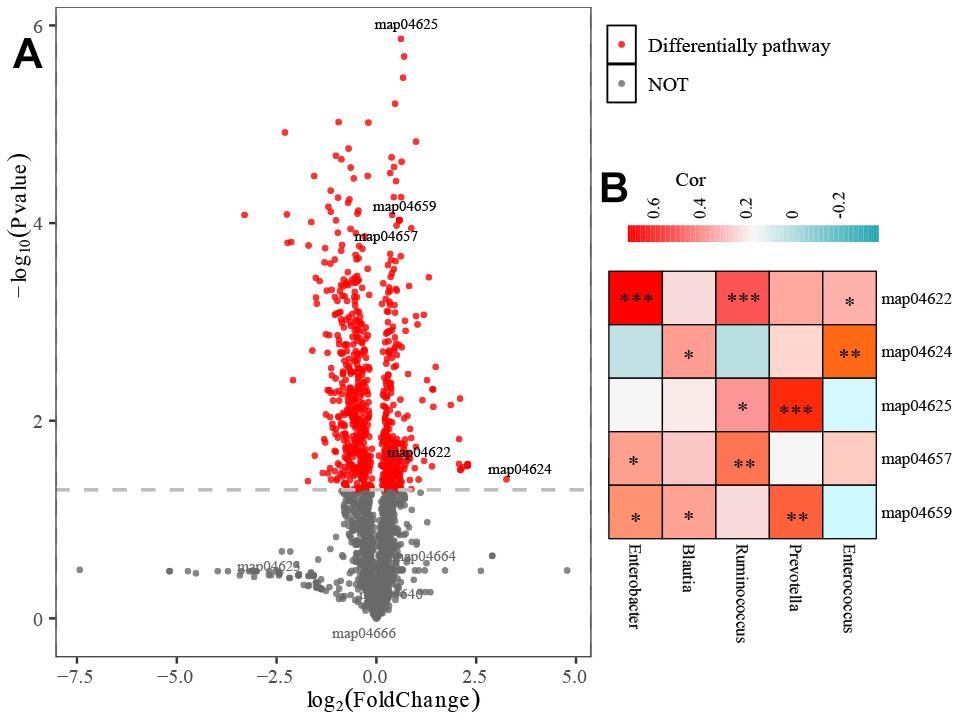
**Functional annotations analysis of gut microbiota.** (**A**) 145 differential pathways identified between H and COPD groups. (**B**) The association of 5 biomarker’s taxa with 5 immune-related pathways.

As shown in Figure 6A, 145 differential pathways were identified between the HC and COPD groups. Of note, 5 immune-related pathways (map04659: Th17 cell differentiation; map04657: IL-17 signaling pathway; map04625: C-type lectin receptor signaling pathway; map04622: RIG-I-like receptor signaling pathway; map04624: Toll and Imd signaling pathway) were significantly enriched in the COPD group. Then, we investigated the association of 5 biomarker’s taxa with 5 immune-related pathways. The Spearman analysis demonstrated that 5 biomarker’s taxa were highly related to immune-related pathways (Figure 6B).

### The profile immunologic factors in COPD

**Figure 7:**
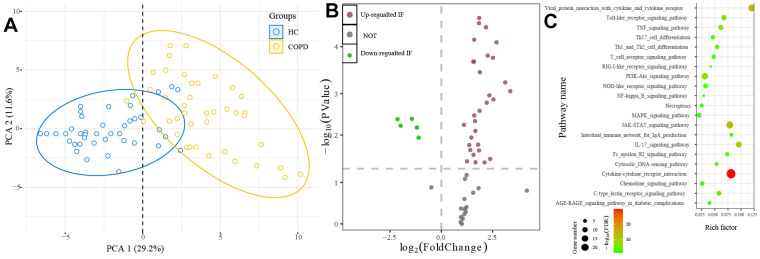
**The profile immunologic factors in COPD.** (**A**) PCA showed that COPD patients and healthy people were gathered in two areas. (**B**) 32 differential IFs were determined between H and COPD group. (**C**) Pathway enrichment analysis of 32 differential IFs. IFs: immunologic factors.

The PCA indicated that the profile of 48 IFs in the COPD group was obviously different from that in the HC group (Figure 7A). The Wilcoxon rank-sum test determined 32 differential IFs, including 27 up-regulated IFs and 5 down-regulated IFs (Figure 7B). Then, we performed pathway enrichment analysis with the online database DAVID (https://david.ncifcrf.gov/home.jsp). The cut-off value was set as FDR <0.05. 21 pathways were enriched by 32 differential IFs (Figure 7C).

### Construction of an IFscore model

**Figure 8:**
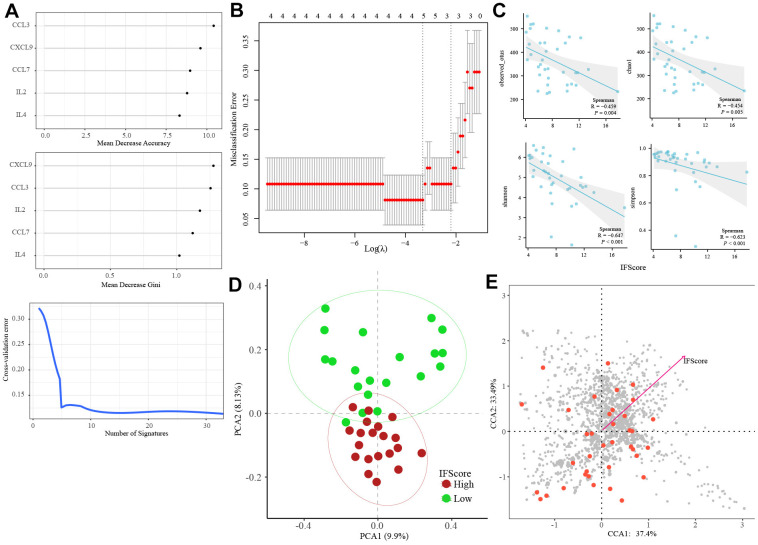
**Construction of IFscore model.** (**A**) Random forest screened out 5 representative IFs with important points. (**B**) LASSO regression analysis was performed to develop a IFscore model with 5 IFs. (**C**) The relation of IFscore to the α gut microbiota. (**D**) The PCA for the β diversity assessment of gut microbiota between high- and low-IFscore samples evidenced that the samples with different IFscore were clustered in different regions. (**E**) The CCA analysis illustrated IFscore had important influence on the distribution and structure of the gut microbiota. LASSO: least absolute shrinkage and selection operator.


Previously, we identified 32 differential IFs. Then, we used the random forest to select 5 representative IFs with important points (CCL3, CXCL9, CCL7, IL2, IL4; Figure 8A). Next, the LASSO regression analysis was performed to develop an IFscore model (Figure 8B). The following formula established the score.

$$\begin{array}{l} IFscore=-9.694+1.433*CCL3+0.029*\text{CXCL}9\\ \;\;\;\;\;\;\;\;\;\;\;\;\;\;\;\;\;\;\;\;+0.001*\text{CCL}7+0.036*\text{IL}2\\ \;\;\;\;\;\;\;\;\;\;\;\;\;\;\;\;\;\;\;\;+0.002*IL4 \end{array}$$

The Spearman analysis indicated that the IFscore was significantly negatively correlated with the observed OTUs (R = -0.459, P =0.004), Chao1 (R = -0.454, P =0.005), Shannon (R = -0.647, P <0.001), and Simpson (R = -0.623, P <0.001) index (Figure 8C). The PCA was utilized for the β diversity assessment of gut microbiota between high- and low-IFscore samples and evidenced that samples with different IFscores were clustered in other regions (Figure 8D). The CCA analysis illustrated that the IFscore was an influential influence factor for the distribution of gut microbiota (Figure 8E).

### Correlation analysis between IFs and biomarker’s taxa

**Figure 9:**
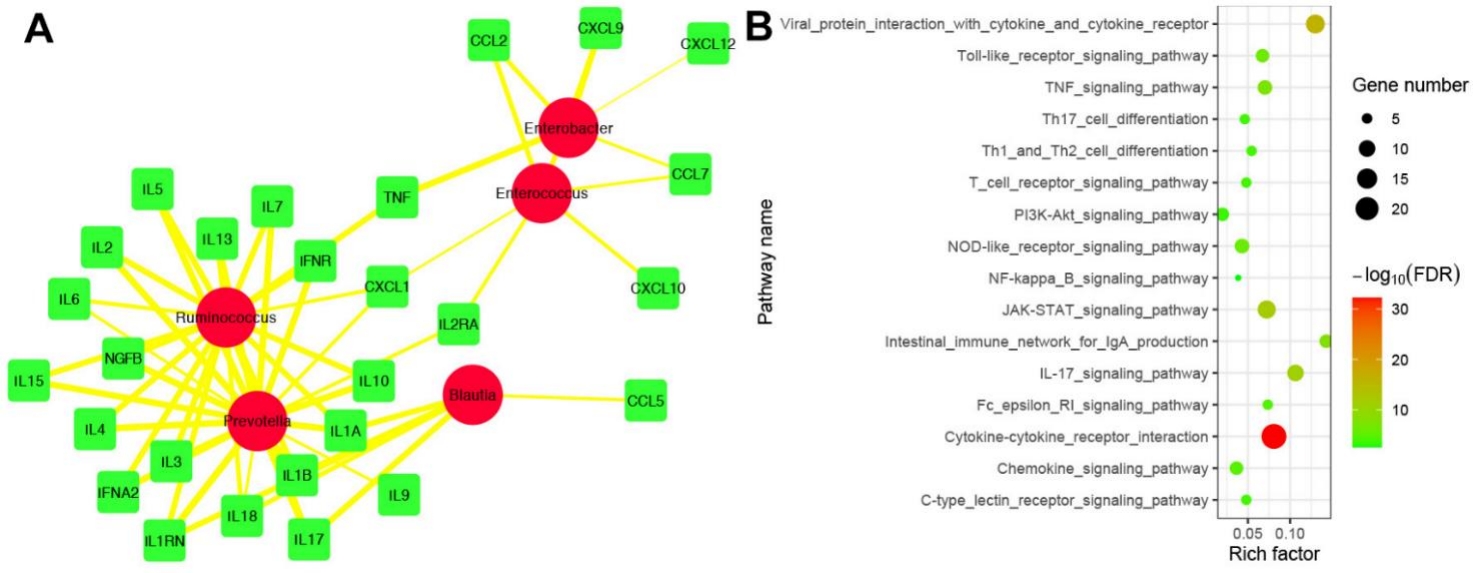
**Correlation analysis between IFs and biomarker’s taxa.** (**A**) The correlation network between IFs and biomarker’s taxa. The red circle represented biomarker’s taxa. The green square represented IFs. The edge represented correlation. The thicker the edge, the greater the correlation. (**B**) Functional enrichment analysis of 29 IFs were highly related to 5 biomarker’s taxa.

The Spearman analysis was applied further to investigate the correlation between IFs and gut microbiota. |cor|> 0.40 and P < 0.05 were set as the cut-off value. 27 IFs were highly related to 5 biomarker’s taxa (Figure 9A). Furthermore, we performed functional enrichment analysis and found that 16 pathways were enriched by 27 IFs (Figure 9B).

## DISCUSSION

In the past decade, a large number of researchers have realized the role of the gut microbiota on the pathophysiological processes of several diseases, including COPD [7]. Recently, multiple studies have observed an imbalance in gut microbiota between COPD patients and healthy individuals, which was in line with our findings. In the study, the α and β diversity revealed that the gut microbiota profile in COPD differed significantly from those in healthy individuals. Previous literature reported that the relative quantity of *Bacteroides* in COPD was higher than that in the healthy, whereas the abundance of *Firmicmicus* was lower [13, 20]. Inconsistent with previous findings, we observed no difference in *Bacteroides* between the two groups, although the proportion of *Firmicmicus* in the COPD group was decreased. A prospective study demonstrated that the gut microbiota was a potential biomarker with clinical validity for the prognostic prediction of COPD [15, 21]. Herein, we also discovered the changes in gut microbiota were closely related to multiple clinical indexes. These results implied that gut microbiota imbalance may contribute to COPD development.

We detected 5 genera as biomarker’s taxa for COPD, including *Blautia, Prevotella, Ruminococcus, Enterococcus*, and *Enterobacter*. *Blautia* is a gram-positive, non-sporulating, coccobacillus shaped bacterium within the guts of mammals and could produce short-chain fatty acids, other organic acids, and H2 and CO2 gases [22]. The abundance of *Blautia* was increased in multiple diseases, and fecal microbiota transplantation would alleviate disease progression [23–26]. *Prevotella* was found conducive to the breakdown of protein and carbohydrates and turned into opportunistic pathogens [27, 28]. *Ruminococcus*, a Gram-positive bacterial, is widely present in the intestine [29]. It is highly related to mucosal inflammation and bile acid metabolism and can improve the content of regulatory T cells and the production of short-chain fatty acids [30, 31]. *Enterococci* are Gram-positive cocci that occur singly, in pairs, or in short chains and have a fermentative metabolism in which they convert carbohydrates to lactic acid [31, 32]. *Enterobacter* bacteria are motile, rod-shaped cells and possess peritrichous flagella. As facultative anaerobes, some *Enterobacter* bacteria ferment glucose and lactose as a carbon source [33]. The levels of *Enterobacter* were found to be significantly higher in patients with COPD, asthma, and lung cancer [14, 34].

Chronic inflammation and immune dysregulation are essential characteristics of COPD and could trigger COPD and accelerate its development. Herein, we investigated the profile immunologic factors in COPD and pinpointed 32 differential IFs (up-regulated 27 IFs and down-regulated 5 IFs). Then, we developed a IFscore with 5 IFs (CCL3, CXCL9, CCL7, IL2, IL4), representing the immune status. CCL3 belongs to the CC chemokine subfamily. The expression of CCL3 under normal physiological conditions is low. However, in COPD patients, immune cells like macrophages, lymphocytes, and neutrophils could secrete and increase the level of CCL3 to participate in immune and inflammatory responses [35]. The inhibition of CCL3 expression could restore the tight junctions of epithelial cells and suppress COPD development [36]. CCL7 is a chemotactic factor for monocytes and neutrophils and is induced in several cell types, like monocytes, vascular smooth muscle cells, and endothelial cells [37]. Decreased CCL7 levels may cause the loss of chemotactic effects on immune cells and a subsequent reduction in inflammatory cell recruitment, while increased CCL7 levels may lead to inflammation [38]. Similar to CCL3, the CCL7 level in COPD patients was enhanced [38]. CXCL9 is secreted by several immune and non-immune cells, including T lymphocytes, eosinophils, macrophages, dendritic cells, fibroblasts, tumor cells, and endothelial cells [39]. CXCL9 induced by IFN-γ functions as a T-cell chemoattractant and can be considered a biomarker of host immune response, especially in Th1 cells mediated immune response [40]. In COPD, the CXCL9 level was increased [40]. In addition, the CXCL9 levels could predict the 12-month COPD-related readmission rate [41]. IL-2 predominantly secretes activated T-cells in an autocrine and paracrine manner to stimulate T-cell differentiation and proliferation and promote host immunity [42]. IL-2 levels were enhanced in COPD patients compared with healthy individuals [43, 44]. IL-4 has the capacity to induce B cell proliferation and Th2 differentiation and switch the immunoglobulin (Ig) class of IgE and IgG4 to play a critical role in inflammation and infection [45]. COPD patients had higher levels of IL-4 than those in healthy individuals. Furthermore, IL-4 level was related to the severity of COPD and could be a potential clinical evaluation marker [46–48].

The gut microbiota can interact with the lungs through the lung–gut axes, which play a causal role in the development and progression of COPD [11]. Nevertheless, the underlying mechanism remains unclear. Herein, we analyzed the correlation between IFs and gut microbiota and identified 29 IFs that were highly related to 5 biomarkers’ taxa. Moreover, we found 29 IFs, especially the pathway, enriched 16 immune-related pathways: cytokine- cytokine receptor interaction, indicating that gut microbiota may influence the development of COPD by regulating the level of cytokine. However, the results still needed more basic experiments to verify.

Limitations: 1. Herein, we unpacked the relation of gut microbiota to IFs. All conclusions were based on high-throughput research and correlation analysis. No experimental research was conducted to confirm the results, and further experiment was demanded. 2. We analyzed the composition and distribution of gut microbiota in COPD patients and selected those 5 genera as the biomarker’s taxa. The included sample size was only 40 cases, which may bring biased results. Therefore, external data was needed to validate the conclusion. 3. We built an IFscore to assess the immune status. However, only 40 samples were used to construct the IFscore model and 0 cases to validate. Hence, large-scale clinical trials were needed to validate the results.

Together, we detected the gut microbiota profile in COPD patients and identified 5 biomarker’s taxa. Additionally, we analyzed the abundance of 48 IFs and determined 32 differential IFs. Then, an IFscore model was developed to evaluate the immune status in COPD patients. Finally, we estimated the association of biomarker’s taxa and IFs and the association of IFscore and the diversity of gut microbiota to conclude that gut microbiota was highly related to the immune status in COPD patients.

## MATERIALS AND METHODS

### Subjects

80 individuals were recruited from Guangfu Hospital, including 40 COPD patients and 40 healthy controls (HC). The study was approved by the medical ethics committee of the Guangfu Hospital, and all participants gave written informed consent. The corresponding clinical information was also collected, such as age, gender, height, weight, body mass index (BMI), smoking history, Global Initiative for Chronic Obstructive Lung Disease (GOLD) Grade, mMRC, COPD Group, Blood Eosinophil, FVC, FEV1, FEV1%pre, and FEV1/FVC. All participants’ detailed demographic and baseline characteristics were described in Table 1.

**Table 1:** The detailed demographic and baseline characteristics of all participants.

	**H (n = 40)**	**COPD (n = 40)**	**P**
Age	68.3 ± 7.34	69.5 ± 7.58	0.674
Gender			0.465
Female	9 (22.5%)	8 (20.0%)	
Male	31 (77.5%)	32 (80.0%)	
Height (cm)	165 ± 7.24	164.5 ± 7.18	0.563
Weight (kg)	57.4 ± 11.54	59.6 ± 12.68	0.742
BMI	21.1 ± 3.34	22.0 ± 3.91	0.683
Smoking history			-
Never	-	11 (27.5%)	
Ever	-	21 (52.5%)	
Current	-	8 (20.0%)	
Frequency of hospitalization ^a^			-
≥1	-	23 (57.5%)	
<1	-	17 (42.5%)	
GOLD Grade			-
I	-	2 (5.0%)	
II	-	15 (37.5%)	
III	-	21 (52.5%)	
IV	-	2 (5.0%)	
mMRC			-
I	-	11 (27.5%)	
II	-	17 (42.5%)	
III	-	6 (15.0%)	
IV	-	6 (15.0%)	
COPD Group			-
A	-	11 (27.5%)	
B	-	6 (15.0%)	
E	-	23 (57.5%)	
Blood Eosinophil (10*9)	-	0.17 ± 0.28	-
FVC	-	2.24 ± 0.61	-
FEV1	-	1.28 ± 0.51	-
FEV1%pre	-	50.66 ± 16.70	-
FEV1/FVC	-	56.16 ± 10.75	-

Notes: ^a^ Frequency of hospitalization due to COPD in the past year. BMI, body mass index.

Patients were diagnosed with COPD according to the GOLD recommendations (2023): FEV1%pre < 80% and FEV1/FVC < 0.7 (after bronchodilators, in a clinically stable condition).

Inclusion criteria in the study: 1) Aged from 18 to 75 years; 2) Meet diagnostic criteria; 3) During the stable period of the condition (>4 weeks after the condition worsens).

Exclusion criteria: 1) With other diseases that would affect the composition and distribution of gut microbiota, like cancer, metabolic diseases, and mental illness; 2) Any antibiotics used intravenously or orally in the past 4 weeks; 3) Women were in pregnancy or breastfeeding.

### Sample collection

The Fecal Collection Kit (Beyotime, China) was used for gathering stool samples. Total genome DNA was extracted using the CTAB method and stored at −80° C. 5 mL blood samples were collected with an EDTA tube and centrifuged (3500 rpm, 4° C, 15 mins). The supernatant was collected as the plasma samples and stored at − 80° C.

### 16s-rRNA gene sequencing

As described previously [49], 1% agarose gel electrophoresis was used to monitor the purity and concentration of DNA. Appropriate samples were taken and diluted to 1ng/μl with sterile water. The primers that targeted the16s-rRNA V3-V4 region was: 341F (5’-CCTAYGGGRBGCASCAG-3’); 806R (5’-GGACTACNNGGGTATCTAAT-3’). The PCR amplification and purification were performed as described previously [49]. Sequencing library construction was performed with NEB Next ® The Ultra DNA Library Prep Kit (Illumina, USA), and the constructed library was tested and quantified using Agilent 5400 (Agilent, USA). After the library was qualified, the library was sequenced on a NovaSeq platform (Illumina, USA), and 250 bp paired-end reads were generated as FASTQ files. Briefly, after format conversion, the plugin dada2 in QIIME2 was used to filter quality, trim, de-noise, merge sequences, and chimerism, and generate the amplicon sequence variant feature table (ASV). The plugin feature-classifier was applied to match ASV to the GREENGENES database to generate the taxonomy table. In addition, the Kyoto Encyclopedia of Genes and Genomes (KEGG) profiles of microbial communities were predicted with PICRUSt (v1.1.2).

### Bio-Plex pro human cytokine 48-plex screening panel

The plasma sample was centrifuged (1000g, 15 min, 4° C) to remove particulates and mixed with wash buffer in a ratio of 1:4. The standards were added 250 μl of standard diluent HB, vortexed 5s and incubated 30 mins. Prepared a fourfold standard dilution series and blank sample. Each well was added 50 μl 1x beads and washed 2 times with 100 μl Bio-Plex Wash Buffer. Then, added 50 μl standards, a plasma sample, blank to well and incubated on shaker at 850 rpm for 30 mins. After washing 3 times, add 25 μl 1x detection antibody and incubate 30 mins. After washing 3 times, added 50 μl 1x streptavidin-PE and incubated for 10 mins. After washing 3 times, resuspended beads in 125 μl assay buffer and generated data with Bio-Plex 3D (Bio-Rad, USA). The data were analyzed using MILLIPLEX Analyst software (V5.1). The fluorescence value of the standard sample was used to obtain the fitting curve, Coefficient of variation (CV), Accuracy, and Sensitivity with the 5-parameter logistic method. Calculated the concentration of immunologic factors by substituting the sample’s fluorescence value into the fitting curve.

### Statistical analysis

The categorical data were presented with No (%) and compared with the Chi-square test. The measurement data were presented with mean ± standard deviation (SD) and compared with the Wilcoxon rank-sum test or one-way ANOVA test. The α diversity was applied to assess the evenness and richness of the gut microbiota presented with observed OTUs, Chao1, Shannon, and Simpson index. The β diversity was utilized to evaluate the extent of the similarity pictured with Principal Component Analysis (PCA), Principal coordinate analysis (PCoA), and Non-metric multidimensional scaling (NMDS) based on Bray–Curtis dissimilarity. The Linear discriminant analysis (LDA) effect size (LefSe) was applied to determine biomarker’s taxa using the Galaxy online platform (http://huttenhower.sph.harvard.edu/galaxy/). LDA value> 3.0 and Wilcoxon rank-sum test: P< 0.01 were set as the threshold. The Spearman analysis was performed to investigate the relationship between the two indexes. The canonical correspondence analysis (CCA) was used to explore the influence factor of the distribution of gut microbiota. The Random forest and least absolute shrinkage and selection operator (LASSO) regression analysis were with packages “randomForestSRC” and “glmnet,” respectively, to screen IFs and develop an IFscore model. All statistical analysis was performed with R 4.1.1 (https://cran.r-project.org) and Prism 7.
